# A Key Role for E-cadherin in Intestinal Homeostasis and Paneth Cell Maturation

**DOI:** 10.1371/journal.pone.0014325

**Published:** 2010-12-14

**Authors:** Marlon R. Schneider, Maik Dahlhoff, David Horst, Benjamin Hirschi, Konrad Trülzsch, Josef Müller-Höcker, Roger Vogelmann, Michael Allgäuer, Markus Gerhard, Sylvia Steininger, Eckhard Wolf, Frank T. Kolligs

**Affiliations:** 1 Institute of Molecular Animal Breeding and Biotechnology, and Laboratory for Functional Genome Analysis (LAFUGA), Gene Center, University of Munich, Munich, Germany; 2 Institute of Pathology, University of Munich, Munich, Germany; 3 Department of Medicine II, Klinikum Grosshadern, University of Munich, Munich, Germany; 4 Institute of Microbiology, Pettenkofer Institute, University of Munich, Munich, Germany; 5 Department of Medicine II, Technical University of Munich, Munich, Germany; Technische Universität München, Germany

## Abstract

**Background:**

E-cadherin is a major component of adherens junctions. Impaired expression of E-cadherin in the small intestine and colon has been linked to a disturbed intestinal homeostasis and barrier function. Down-regulation of E-cadherin is associated with the pathogenesis of infections with enteropathogenic bacteria and Crohn's disease.

**Methods and Findings:**

To genetically clarify the function of E-cadherin in intestinal homeostasis and maintenance of the epithelial defense line, the *Cdh1* gene was conditionally inactivated in the mouse intestinal epithelium. Inactivation of the *Cdh1* gene in the small intestine and colon resulted in bloody diarrhea associated with enhanced apoptosis and cell shedding, causing life-threatening disease within 6 days. Loss of E-cadherin led cells migrate faster along the crypt-villus axis and perturbed cellular differentiation. Maturation and positioning of goblet cells and Paneth cells, the main cell lineage of the intestinal innate immune system, was severely disturbed. The expression of anti-bacterial cryptidins was reduced and mice showed a deficiency in clearing enteropathogenic bacteria from the intestinal lumen.

**Conclusion:**

These results highlight the central function of E-cadherin in the maintenance of two components of the intestinal epithelial defense: E-cadherin is required for the proper function of the intestinal epithelial lining by providing mechanical integrity and is a prerequisite for the proper maturation of Paneth and goblet cells.

## Introduction

The intestinal epithelial lining is subject to continuous chemical, physical and biological insults. Therefore, an integral component of intestinal homeostasis is maintenance and repair of the epithelial barrier itself. This barrier is constituted by three main components: The intestinal epithelial cells themselves, mucins and antibacterial products secreted by these, and the adaptive immune response [1]. The gastrointestinal epithelial lining consists of a monolayer of cells that undergoes rapid and continuous self-renewal from the base of the crypts, where multipotent stem cells reside [2], [3]. The small intestinal epithelium is composed of four distinct differentiated cell types: absorptive enterocytes, mucus-producing goblet cells, hormone-secreting enteroendocrine cells, and antibacterial peptide-secreting Paneth cells which in contrast to the other cell lineages remain at the crypt base. The continuous production of new cells is balanced by apoptosis at the luminal side, resulting in a cellular turn over rate of three to five days in the mouse.

Intercellular junctions are a major prerequisite for tissue integrity and tissue polarization. The apical junctional complex of the intestinal epithelium is constituted by tight junctions, adherens junctions, and desmosomes [4]. Tight junctions are continuous, circumferential belt-like structures that form a permeability barrier at the apical end of the intercellular space. Adherens junctions reside immediately subadjacent to tight junctions and play an important role in cell recognition and in mediating intercellular associations. Desmosomes, which are located below adherens junctions, are spot-like intercellular junctions. Especially in stratified epithelia like the epidermis they provide strong intercellular association [5].

The major constituent of adherens junctions is E-cadherin. E-cadherin forms homophilic cell-cell interactions and intracellularly binds to catenins (β-catenin, plakoglobin, p120-catenin) which link the transmembranous E-cadherin via α-catenin to the actin cytoskeleton [6]. E-cadherin elicits many functions in tissue morphogenesis and is essential in embryo development [7]. Loss of E-cadherin function in the intestine has been linked to pathological processes. Several studies have reported reduced expression of E-cadherin in inflamed epithelium of patients with Crohn's disease and ulcerative colitis [8]–[10]. Especially in Crohn's disease the altered epithelial barrier is believed to be a primary factor in the development of the disease [11]. Recently, polymorphisms in the *CDH1* gene resulting in truncated and intracellularly mis-localized E-cadherin have been identified in patients with Crohn's disease [12]. Moreover, during progression of colorectal and other tumors a switch in cadherin expression from E-cadherin to N-cadherin is observed coinciding with the transition from an epithelial to a mesenchymal phenotype leading to an increase in the invasive capabilities of cancer cells [6] and inactivation of one E-cadherin allele enhances tumor initiation in mice carrying a mutated adenomatous polyposis coli (APC) gene [13].

To date E-cadherin has not been directly targeted in the mouse intestine to clarify its role in this tissue. Indirect data generated by over-expression of a dominant-negative N-cadherin or targeting p120-catenin suggest an important role in intestinal homeostasis [14], [15]. To genetically clarify the role of E-cadherin in the homeostasis of the intestinal epithelium, we inactivated the *Cdh1* gene in the mouse small intestine and colon by using the Cre-LoxP system. We report here that loss of E-cadherin expression results in loss of adherens junctions and desmosomes, leading to apoptosis and shedding of cells. Moreover, positioning and maturation of Paneth and goblet cells is severely impaired and the number of goblet cells is reduced. This results in the deficiency in clearing pathogenic bacteria from the intestinal lumen. Our data clarify E-cadherin's essential role in the homeostasis of small and large intestine and its critical contribution to the intestinal epithelial defense line.

## Materials and Methods

### Ethics Statement

The maintenance and breeding of mouse lines and all experiments were approved by the Committee on Animal Health and Care of the local governmental body of the state of Upper Bavaria (Regierung von Oberbayern; TA087/09) and performed in strict compliance with the EEC recommendations for the care and use of laboratory animals (European Communities Council Directive of November 24, 1986 [86/609/EEC]).

### Animals

Mice homozygous for the floxed E-cadherin allele *Cdh1^fl/fl^* [16] were bred with animals carrying the *Villin-Cre-ER^T2^* transgene [17], litters were genotyped by PCR as described in the original publications. Subsequently, double heterozygous *Villin-Cre-ER^T2^;Cdh1^wt/fl^* animals were mated with *Cdh1^fl/fl^* animals to generate *Villin-Cre-ER^T2^;Cdh1^fl/fl^* mice. From the same crossings, animals carrying one wild-type E-cadherin allele and mice lacking the Cre transgene were obtained which served as control animals. All mice were in the C57BL/6 background. Animals were maintained under specific pathogen-free conditions in a closed barrier system and had free access to a standard rodent diet (V1534, Ssniff, Soest, Germany) and water.

Bioluminescent yersiniae were constructed by integrating the LuxCDABE operon under the L-arabinose-inducible araBAD promoter (PBAD) downstream of glmS in *Yersinia enterocolitica* WA-C(pYV::Cm) as described previously [18]. Six- to 8-week-old female Cre^+^ *Cdh1^fl/fl^* and control mice (Cre^+^ *Cdh1^wt/fl^*) were infected orally with the bioluminescent yersiniae [18] from frozen stock suspensions. These were prepared by growth to stationary phase in LB medium at 27°C and freezing the bacteria in 15% glycerol at −80°C. After appropriate dilutions, bacteria were washed twice with phosphate-buffered saline and mice were fed 50 µl of the solution containing 10^9^ CFU with a pipette to the back of the oropharynx after fasting for 16 h. To induce luminescence of yersiniae, mice were intraperitoneally injected with 120 mg L-arabinose in PBS as described previously [18]. Detection of luminescence was performed with the IVIS Lumina System (Caliper Life Sciences, Mainz, Germany).The levels of colonization of Peyer's patches and small intestinal lumina were determined by plating of serial dilutions onto Mueller-Hinton agar for 36 h at 27°C as described previously [19].

### Induction of recombination and BrdU labeling

4-OH-tamoxifen (Sigma, Deisenhofen Germany) was dissolved in corn oil (Sigma) (final concentration 10 mg/ml). Mice received 1 mg tamoxifen per intraperitoneal injection either for 3 days or 5 days in a row or on days 1, 2, 5, and 8. Mice were sacrificed for analysis on days 4, 6 or 12, respectively, after start of tamoxifen injections. To analyze cell proliferation in the intestine, animals received a single intraperitoneal injection of BrdU (Roche Diagnostics, Mannheim, Germany; 30 mg/kg body weight) 1 hour or 24 hours prior to sacrifice.

### Tissue processing, immunohistochemistry, immunofluorescence and electron microscopy

At different time points after tamoxifen injection, anaesthetized mice were sacrificed by exsanguination under isoflurane anesthesia. The intestine was removed immediately, carefully trimmed free of adjacent tissues, and cleared of stool. The intestines were fixed in 4% paraformaldehyde (in PBS) in the form of “swiss rolls” [20], dehydrated and embedded in paraffin. From the paraffin-embedded specimens, serial sections were cut and stained with hematoxylin and eosin (H&E). Periodic acid-Schiff reagent (PAS) and Alcian Blue (AB) staining were performed according to standard protocols. Immunohistochemical staining was performed on 5 µm sections of paraformaldehyde fixed, paraffin embedded tissue samples. The primary antibodies were rat anti-BrdU (Oxford Biotechnology, Kidlington, UK), rat anti-mouse Ki67 (Dako, Hamburg, Germany), goat anti-E-cadherin (R&D Sytems, Wiesbaden, Germany), rabbit anti-cleaved caspase 3 (Cell Signaling, Frankfurt, Germany), rabbit anti-chromogranin A (Immuno Star, Hudson, USA), goat anti-EphB3 (R&D Systems), goat anti-villin (Santa Cruz Biotechnology, Wiesbaden, Germany), rabbit anti-lysozyme (Dako), mouse anti-β-catenin (BD Biosciences), goat anti-MMP7 (R&D Systems). Isotype controls were run for every antibody on parallel sections. For antigen unmasking, sections were either sub-boiled for 20 min at 95°C in 10 mM sodium citrate buffer (pH 6.0, 10mM; for BrdU, Ki67, E-cadherin,β-catenin, MMP7, and cleaved caspase 3) or treated with Target Retrieval Solution (Dako) (for EphB3), with Epitope Retrieval Solution (Novocastra Laboratories, Newcastle upon Tyne, UK) (for chromogranin A), with Target Unmasking fluid (Vector Laboratories, Burlingame, USA) (for Villin) or with Proteinase Type XXIV (Novocastra) (for lysozyme). Antigen detection was performed with the ImmPRESS Reagent Kit Anti-Rabbit or Anti-Goat (Vector Laboratories) or with the Vectastain ABC-Kit Elite Rat or Goat IgG (Dako). AEC (Zymed Systems, Berlin, Germany) or DAB (Dako) were used as chromogen. Slides were finally counterstained with hematoxylin (Vector Laboratories). For the preparation of semi-thin sections azur-methylenblue staining was applied. For electron microscopy freshly prepared segments of small intestine were fixed in 6.25% glutaraldehyde/Soerensen phosphate buffer for 2 hours. After washing in buffered saccharose and osmication (2% distilled water) for 1 hour, tissues were dehydrated in acetone and then processed for Epon embedding (polymerisation at 78°C overnight). Ultrathin sections were counterstained with uranyl and lead citrate. Samples were studied with a Philips EM 420 transmission electron microscope.

For confocal immunofluorescence microscopy, mouse tissues were fixed with 2% paraformaldehyde in 100mM sodium phosphate buffer, pH 7.4, dehydrated (15% sucrose followed by 30% sucrose) and embedded in Tissue Tek (Sakura Finetek, Zoeterwoude, The Netherlands). Collagen of tissue slices (16 µm) was quenched (75 mM NH4Cl, 20mM Glycin in phosphate-buffered saline, PBS) followed by permeabilization in PBS+3% bovine serum albumin+1% saponin+0.1% Triton X for 30 min at room temperature, respectively. Tissues were stained with antibodies against lysozyme (anti-goat, Santa Cruz Biotechnology, Heidelberg, Germany), Muc2 (anti-rabbit, Santa Cruz Biotechnology), E-Cadherin (Alexa Fluor 647, anti-mouse, BD Biosciences), and β-catenin (Alexa Fluor 546 nm, anti-mouse, BD Biosciences) overnight at 4°C. Secondary antibodies (anti-goat Alexa Fluor 488 and anti-rabbit Alexa Fluor 546) were from Invitrogen (Karlsruhe, Germany). Samples were embedded in vectashield with DAPI (Vector Laboratories) and imaged with a Leica SP5 confocal microscope (Wetzlar, Germany), and z-stacks were projected onto three-dimensional reconstructions using Volocity 4.1 software (Perkin Elmer, Waltham, CA, USA). Figures were assembled with Photoshop CS (Adobe Systems, Munich, Germany).

### *In-situ* hybridization

Probes for murine Axin2 were generated from EST clone, corresponding to nucleotides 3472–4256 of the murine Axin2 cDNA (NM_015732.4). In vitro transcription reactions were performed using 1 µg of linearized DNA, 2 µl of Dig RNA labeling mix (Roche), 1 µl of ribonuclease inhibitor (Promega, Mannheim, Germany), and 1.5 µl of T7, T3, or SP6 (Promega, Germany). In-situ hybridization was performed essentially as described previously [21].

### Quantitative RT-PCR

Total RNA was isolated from intestinal mucosa with Trizol (Invitrogen) and reverse transcribed using the SuperScript First Strand kit (Invitrogen). Quantitative RT-PCR was performed in a Rotorgene 3000 thermocycler (Corbett Life Science, Sydney, Australia) using primers for cryptidin 1 and 4, Muc2, lysozyme and GAPDH primer pairs (Metabion, Martinsried, Germany) and SYBR Green I (Invitrogen). The oligonucleotide sequences used were: GAPDH, forward, 5′-TCATCAACGGGAAGCCCATCAC-3′, reverse, 5′-AGACTCCACGACATACTCAGCACCG-3′; cryptidin 1 (Defcr1), forward, 5′-TCAAGAGGCTGCAAAGGAAGAGAAC-3′, reverse, 5′-TGGTCTCCATGTTCAGCGACAGC-3′; cryptidin 4 (Defcr4), forward, 5′-CCAGGGGAAGATGACCAGGCTG-3′, reverse, 5′-TGCAGCGACGATTTCTACAAAGGC-3′. Muc2, forward, 5′-ACAAAAACCCCAGCAACAAG-3′, reverse, 4′-RV GAGCAAGGGACTCTGGTCTG-3′; lysozyme, forward, 5′-GCCAAGGTCTACAATCGTTGTGAGTTG-3′, reverse, 5′-CAGTCAGCCAGCTTGACACCACG-3′. Differential expression of mRNAs was calculated using the ΔΔCt method.

## Statistical analysis

Groups were compared using two-tailed Student's t-test and a two-tailed Mann-Whitney test (Prism 4, GraphPad Software, La Jolla, CA) and p<0.05 was considered significant.

## Results

### E-cadherin is required for the maintenance of the intestinal epithelial architecture

**Figure 1:**
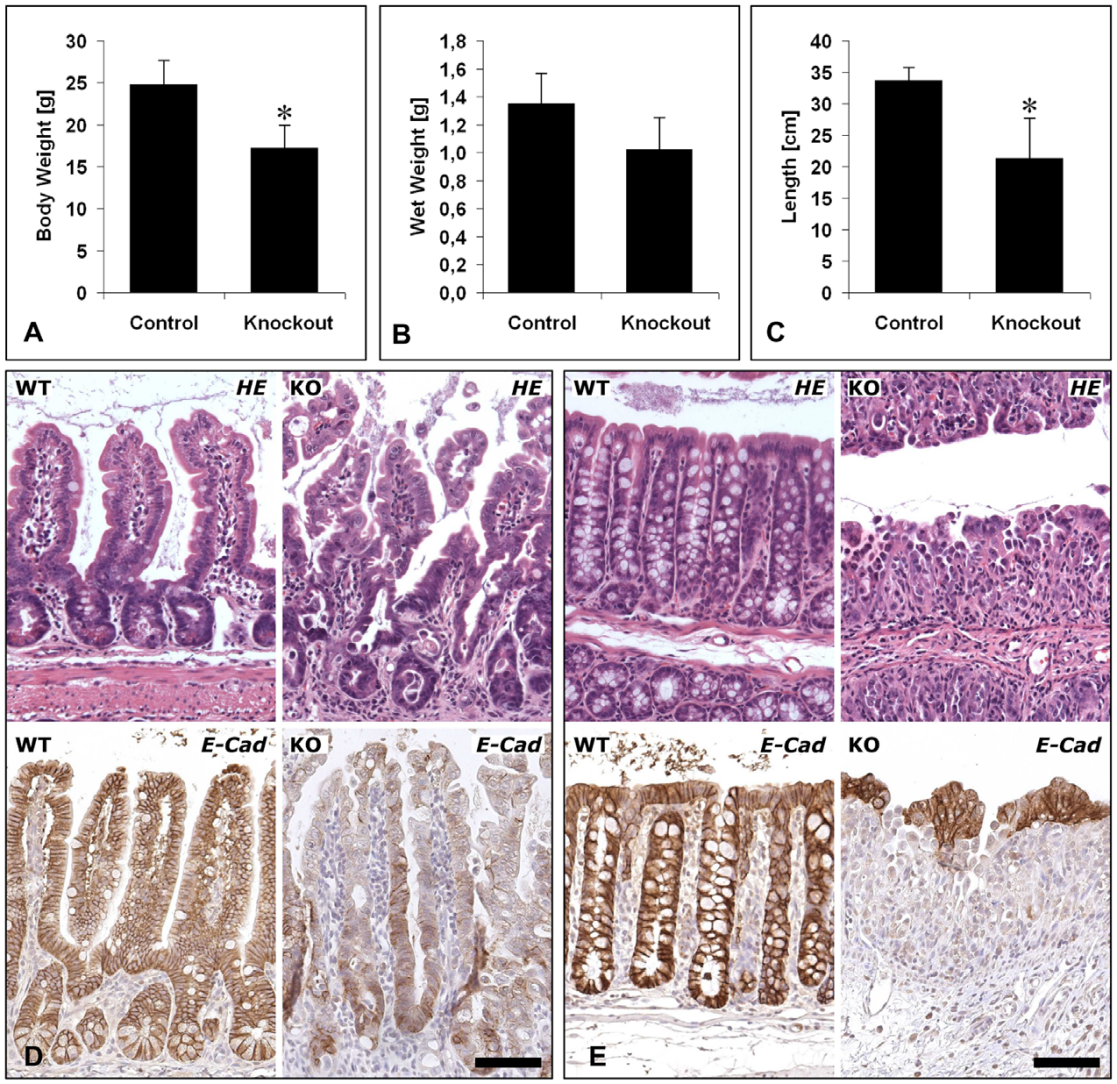
E-cadherin is required for the maintenance of the intestinal epithelial architecture. (A) Deletion of E-cadherin results in severe loss of body weight in Cre^+^ *Cdh1* ^fl/fl^ mice (n = 4) as compared to gender-matched littermates (n = 6) (*P*<0.005). Necropsy revealed a reduction of (B) wet weight (*P* = 0.066) and (C) the length of small intestine (*P* = 0.027). (D and E, upper panels). Comparison of H&E-staining of tamoxifen-treated control mice (WT) and Cre^+^ *Cdh1* ^fl/fl^ (KO) mice revealed a severe disruption of the epithelial architecture of small intestine (D) and colon (E). (D and E, bottom panels) Immunostaining for E-cadherin revealed a strong reduction in E-cadherin expression 6 days after start of recombination. Mean values and standard deviations are shown. Bars, 100 µm.

To investigate the role of E-cadherin for the maintenance of the intestinal epithelial barrier, mice bearing floxed *Cdh1* alleles [16] were crossed into an inducible transgenic background, which uses the villin promoter to deliver tamoxifen-inducible Cre activity in the intestine [17]. Villin-Cre-ER^T2^; *Cdh1* ^fl/fl^ (Cre^+^ *Cdh1* ^fl/fl^) mice and respective control mice (Cre^+^ *Cdh1* ^fl/+^ and *Cdh1* ^fl/fl^) were subjected to tamoxifen injections intraperitoneally at 8–10 weeks of age. Cre^+^ *Cdh1* ^fl/fl^ mice injected for five consecutive days were visibly ill on day 6, presented with hemorrhagic diarrhea and needed to be euthanized. In contrast, control mice including Cre^+^ *Cdh1* ^fl/+^ treated with tamoxifen and untreated Cre^+^ *Cdh1* ^fl/fl^ mice revealed no symptoms of illness. While control animals gained some weight during the six day period, Cre^+^ *Cdh1* ^fl/fl^ mice lost up to 25% of their body weight (Fig. 1A). Moreover, wet weight and length of small intestine were considerably reduced (Fig. 1B, C). Microscopic analysis of H&E-stained sections from the small intestine of Cre^+^ *Cdh1* ^fl/fl^ mice on the sixth day after start of tamoxifen injection revealed that the crypt-villus architecture of Cre^+^ *Cdh1* ^fl/fl^ mice appeared severely altered as compared to Cre^+^ *Cdh1* ^fl/+^ and *Cdh1* ^fl/fl^ mice also treated with tamoxifen (Fig. 1D, upper panels). The phenotype was even more dramatically altered in the colon where the entire crypt architecture was lost (Fig. 1E, upper panels). In induced Cre^+^ *Cdh1* ^fl/fl^ mice, E-cadherin immunostaining was grossly reduced and confined to some cells at the bottom of the crypt in small intestine (Fig. 1D, lower panels) and to a few cells at the apical site of the colon compared to control mice (Fig. 1E, lower panels).

### Induction of cell death and loss of adherens junctions and differentiated cells upon loss of E-cadherin

**Figure 2:**
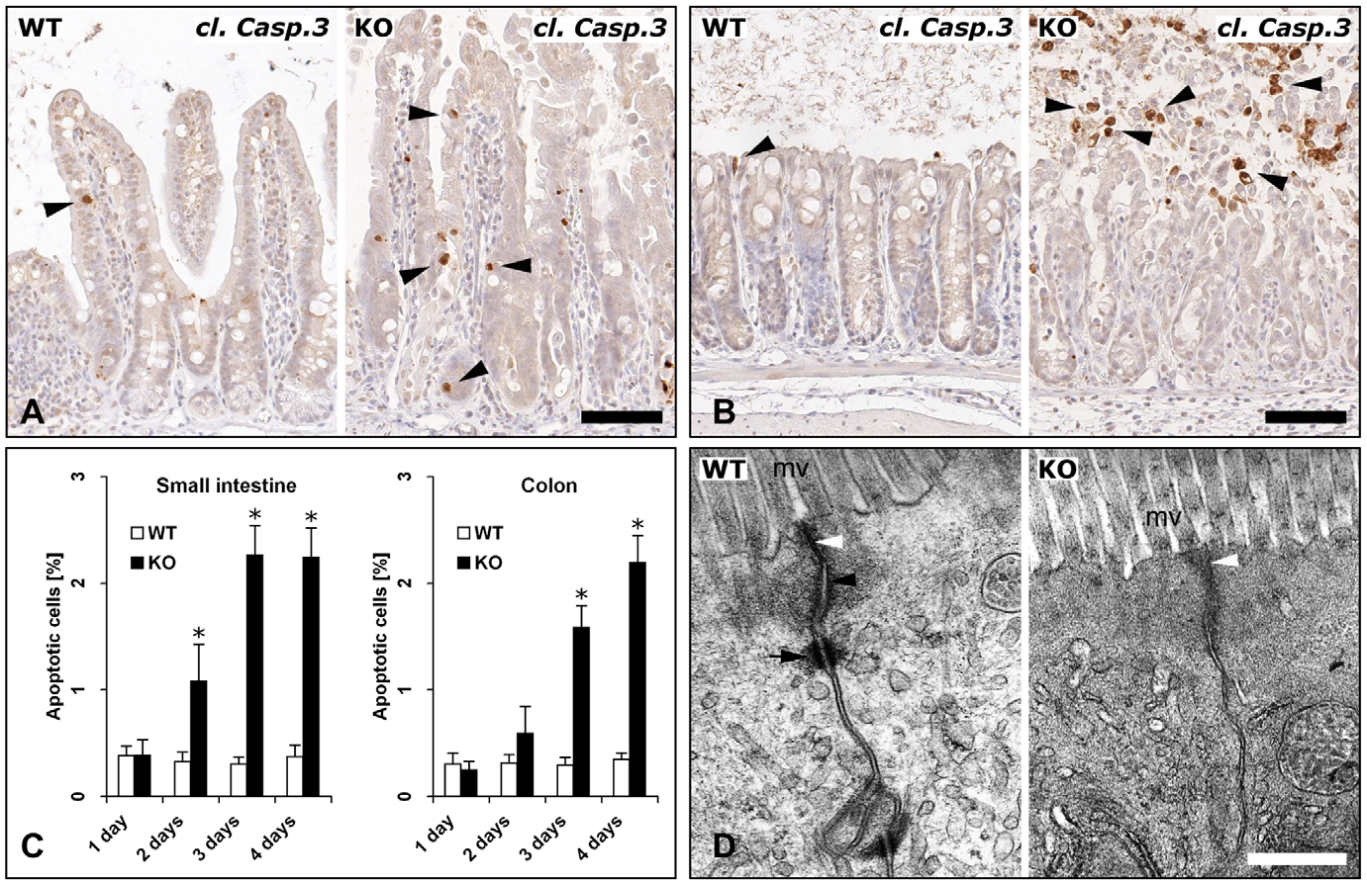
Induction of cell death and loss of adherens junctions and desmosomes upon loss of E-cadherin. (A and B) Immunostaining of small intestine (A) and colon (B) for cleaved caspase 3 in control (WT) and Cre^+^ *Cdh1* ^fl/fl^ (KO) mice treated with tamoxifen for 5 days was employed to identify apoptotic cells (arrowheads). (C) Quantitative analysis of apoptotic cells. 20 crypt-villus units in small intestine and 20 crypts in colon per animal were analyzed at the indicated time points (n = 4 mice/group). The rate of apoptotic cells is expressed as a percentage of total cell numbers and mean values and standard error means are shown (*, *P*<0.005). (D) Transmission electron microscopy of the apical junctional complex of control (WT) and Cre^+^ *Cdh1* ^fl/fl^ (KO) mice. The intercellular space, microvilli (mv) and cellular substructures appeared unaltered. Compared to control mice, the junctional complex of Cre^+^ *Cdh1* ^fl/fl^ mice had preserved tight junctions (white arrowhead), but lacked adherens junctions with *rete terminale* (black arrowhead) and desmosomes (black arrow). Bars in A and C, 100 µm, in D 0.2 µm.

Already on the second day after start of recombination, the number of apoptotic cells was significantly increased in the small intestine of Cre^+^ *Cdh1* ^fl/fl^ mice as compared to control animals (Fig. 2A and C). On the third and fourth days the apoptotic cell count was further increased with apoptotic cells distributed all over the crypts and villi (Fig. 2A). In the colon an increase in apoptosis was observed on the third day (Fig. 2C). On the fifth day, loss of E-cadherin in the colon led to shedding of cell sheets resulting in loss of the cellular surface layer (Fig. 2B). Next, the ultrastructure of cells and cell adhesion molecules was studied by transmission electron microscopy. This revealed no changes in the intercellular space and normal appearing microvilli in wild-type and Cre^+^ *Cdh1* ^fl/fl^ mice (Fig. 2D). Wild-type mice revealed typical apical junctional complexes consisting of tight junctions, adherens junctions, and desmosomes (Fig. 2D, left panel). Tight junctions also appeared unchanged in Cre^+^ *Cdh1* ^fl/fl^ mice treated with tamoxifen for 3 days. However, adherens junctions including the *rete terminale* which represents the connection of the actin cytoskeleton to the E-cadherin adhesional complex and the desmosomes were lost from the majority of cell junctions in recombined mice (Fig. 2D, right panel).

**Figure 3:**
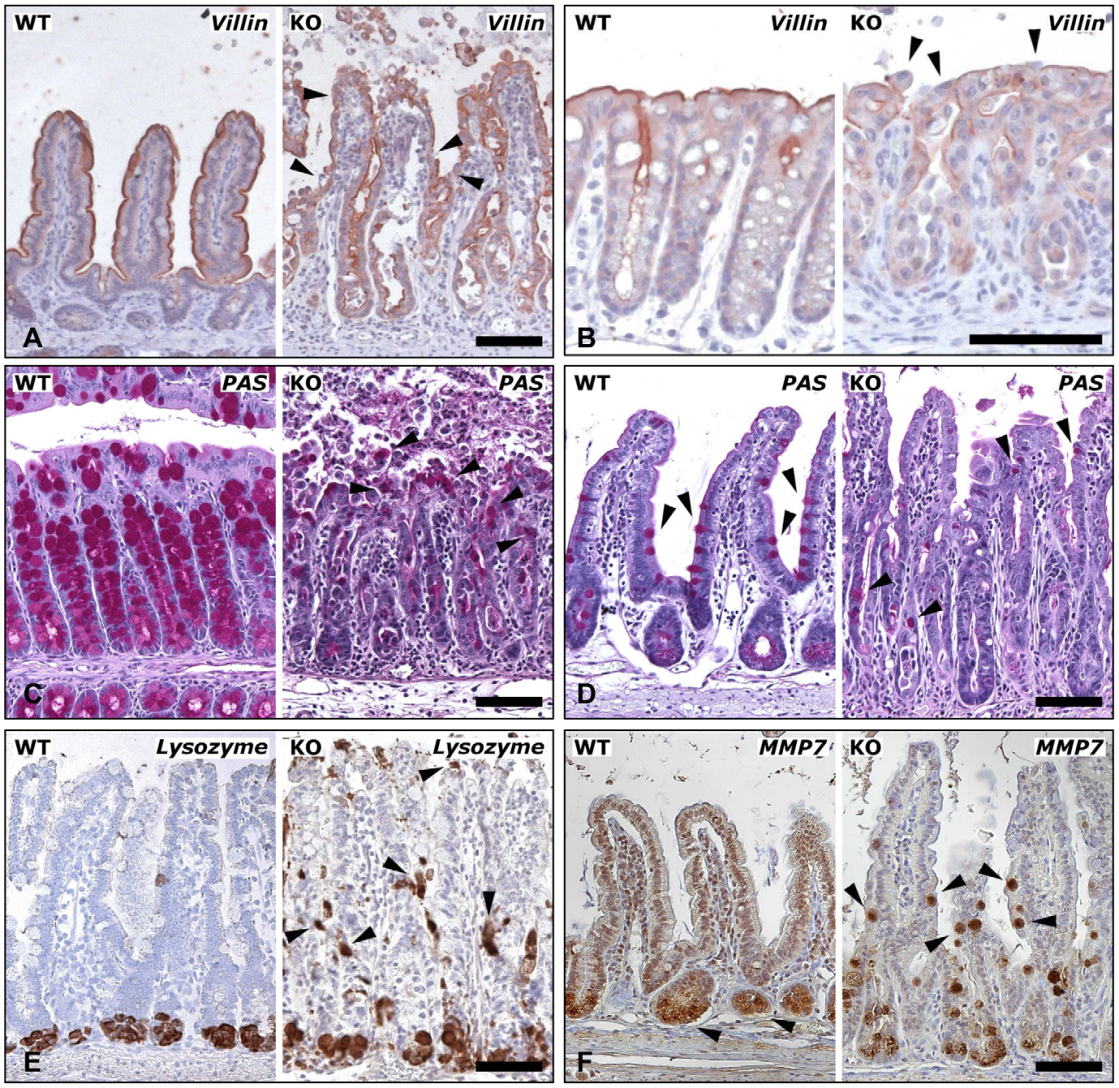
Loss of differentiated cells following loss of E-cadherin. (A and B) Staining of absorptive enterocytes for villin. Reduced staining intensity and disruption of the epithelial lining (arrowheads) in the small intestine (A) and colon (B) of Cre^+^ *Cdh1* ^fl/fl^ (KO) mice as compared to controls (WT). (C and D) PAS staining revealed a strong reduction of goblet cells (arrowheads) in the colon (C) and small intestine (D) of Cre^+^ *Cdh1* ^fl/fl^ mice four days after start of induction of recombination as compared to control mice. (E and F) Paneth cells were identified by immunostaining for lysozyme (E) and MMP7 (F). After five days of tamoxifen treatment, Paneth cells remained confined to the base of the crypt in control mice, but were distributed throughout the crypt-villus axis in Cre^+^ *Cdh1* ^fl/fl^ mice (arrowheads). Bars in A–F, 100 µm.

After 5 tamoxifen injections, expression of villin, a marker for absorptive enterocytes, was confined to the surface area of small intestine and colon in control mice (Fig. 3A and B, left panels). At the same time point, this surface staining was reduced and partly interrupted in recombined Cre^+^ *Cdh1* ^fl/fl^ mice, consistent with a disintegration of the intestinal epithelial lining (Fig. 3A and B, right panels). Next, the secretory cell lineages were studied. Histochemical analysis of the colon and small intestine from induced Cre^+^ *Cdh1* ^fl/fl^ mice with PAS and AB staining revealed that the number of mucus producing goblet cells was strongly reduced (Fig. 3C and 3D, and data not shown). After five days of tamoxifen treatment the ratio of PAS positive cells per total cells in the crypt-villus units of the small intestine was reduced to a mean value of 3.0±0.4% compared to 9.5±0.8% in controls (p<0.001; n = 4 mice per group; 20 crypt-villus units counted per mouse). Paneth cells are unique to the small intestine where they reside at the bottom of the crypts as demonstrated by lysozyme and MMP7 staining (Fig. 3E and F, left panels). After loss of E-cadherin, lysozyme and MMP7 positive cells were no longer confined to this location but were scattered all over the crypts and villi in Cre^+^ *Cdh1* ^fl/fl^ mice (Fig. 3E and F, right panels). Of note, several of the lysozyme and MMP7 positive cells in the villi were considerably larger than the cells at the bottom of the crypts. The number of the scarce enteroendocrine cells, as evaluated by synaptophysin staining, did not appear to be changed in Cre^+^ *Cdh1* ^fl/fl^ mice (data not shown).

### E-cadherin deficiency abrogates localization and maturation of secretory cells

**Figure 4:**
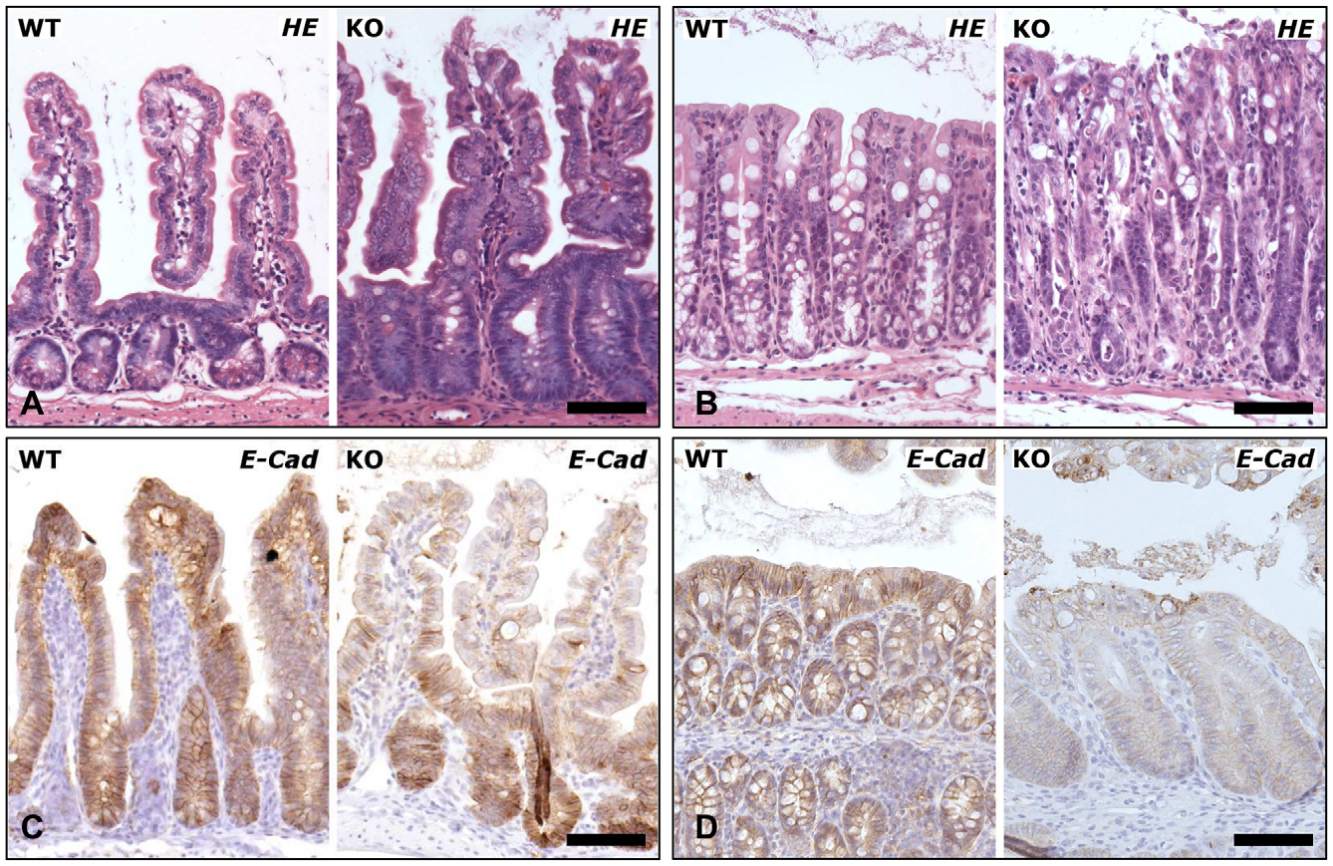
Impaired expression of E-cadherin results in elongation of crypts and villi. H&E staining of Cre^+^ *Cdh1* ^fl/fl^ (KO) and control mice (WT) on day 12 after induction of recombination on days 1, 2, 5, and 8, revealed milder changes in the epithelial architecture of small intestine (A) and colon (B) with elongation of crypts and disorganization of the cellular order. Immunostaining for E-cadherin revealed a reduction in E-cadherin expression 12 days after start of recombination in KO compared to WT mice in small intestine (C) and colon (D). Bars, 100 µm.

**Figure 5:**
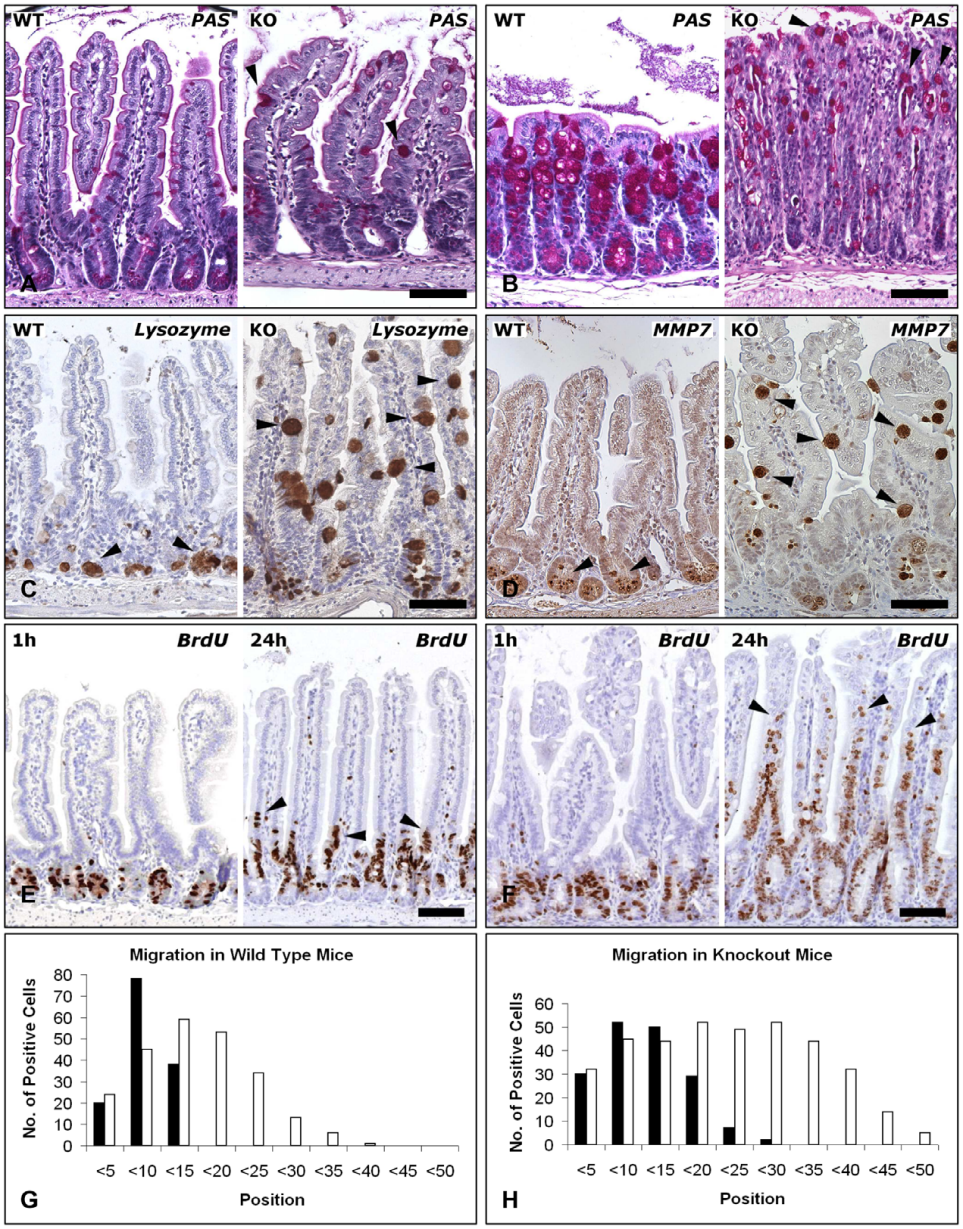
E-cadherin deficiency abrogates localization and differentiation of cells. (A and B) PAS staining of small intestine (A) and colon (B) in control mice (WT) and Cre^+^ *Cdh1* ^fl/fl^ mice (KO) analyzed on day 12 following treatment with tamoxifen on days 1, 2, 5, and 8 revealed a reduction of the number of goblet cells and the appearance of large atypical goblet cells (arrowheads). (C and D) Staining for lysozyme (C) and MMP7 (D) in control mice (WT) and Cre^+^ *Cdh1* ^fl/fl^ mice (KO) analyzed on day 12 following treatment with tamoxifen as described above revealed a reduction of Paneth cells at the base of the crypts and the appearance of large positively stained cells in the villi (arrowheads). (E and F) BrdU staining to evaluate cell migration in control (E) and Cre^+^ *Cdh1* ^fl/fl^ (F) mice 1h (left) and 24h (right) after injection of BrdU. After 24h, BrdU-positive cells were still confined to the lower parts of the villi in control mice, but positive cells were found throughout the entire crypt-villus axis in Cre^+^ *Cdh1* ^fl/fl^ mice. (G and H) Position of BrdU-positive cells at 1 h (black bars) and 24 h (open bars) within the crypt-villus axis of control and Cre^+^ *Cdh1* ^fl/fl^ mice. Position 0 represents the base of the crypt. Data were obtained by evaluating 20 crypt-villus units per mouse (n = 2 animals/group). Bars in A and B, 100 µm.

As the severe changes of the intestinal epithelium caused by loss of E-cadherin were not compatible with life, precluding a more detailed study of E-cadherin's role in differentiation and localization of cells, a milder recombination protocol allowing mice to survive for a longer period was developed. For this purpose, mice were treated with one daily intraperitoneal injection of tamoxifen on days 1, 2, 5, and 8. Cre^+^ *Cdh1* ^fl/fl^ mice treated with this attenuated protocol did not reveal any differences in behavior, stool habits, or body weight when they were sacrificed on day 12. Microscopic analysis revealed a less severe array of pathological alterations in the small intestine and colonic crypts as compared to the lesions observed in Cre^+^ *Cdh1* ^fl/fl^ mice receiving tamoxifen for five days. Crypts were elongated and appeared disorganized (Fig. 4A and B). Expression of E-cadherin was reduced (Fig. 4C and D). The number of goblet cells was again reduced in both small intestine and colon (Fig. 5A and B). In the colon, goblet cells now appeared predominantly in the upper half of the crypts. In addition, several considerably larger goblet cells were found in both the small intestine and colon. Paneth cells in the Cre^+^ *Cdh1* ^fl/fl^ mice were distributed all over the crypts and villi and the number of Paneth cells at the bottom of the crypts appeared reduced (Fig. 5C and D). Moreover, in addition to small cells, several of the lysozyme-positive (Fig. 5C) and MMP7-positive (Fig. 5D) cells in the villi were much larger than typical Paneth cells. These data suggest a perturbation of Paneth and goblet cell maturation and migration after loss of E-cadherin.

As loss of E-cadherin affected localization of differentiated cells, cell migration was studied in more detail. Mice receiving the attenuated recombination protocol were injected with BrdU 1 or 24 h before sacrifice at day 12 to mark cells in the S phase. We found that BrdU-labeled cells in the control mice were exclusively located in the lower parts of the crypts in the 1 h group (Fig. 5E, left panel) and only few cells had migrated towards the villi in the 24 h group (Fig. 5E, right panel). In induced Cre^+^ *Cdh1* ^fl/fl^ mice, analysis of BrdU stained cells identified an expanded proliferative zone in small intestine (Fig. 5F, left and right panels). The mean number of BrdU positive cells in control and in Cre^+^ *Cdh1* ^fl/fl^ mice sacrificed 1 hour after BrdU injection differed significantly (6.8±1.4 and 8.5±1.6 BrdU-positive cells per crypt/villus unit, respectively; p<0.001). After 24 h the difference was increased from 11.8±2.6 in control mice to 18.5±2.8 BrdU-positive cells per crypt/villus unit in Cre^+^ *Cdh1* ^fl/fl^ mice (p<0.001). While a normal cell migration pattern was observed in control mice, with the majority of proliferating cells being localized close to the crypt basis 1 h after BrdU injection with a moderate shift towards the villus 24 h after BrdU injection (Fig. 5G), cell migration was clearly disturbed in Cre^+^ *Cdh1* ^fl/fl^ mice: 24 h after exposure to BrdU, proliferating cells were found almost uniformly distributed along the lower two thirds of the crypt-villus axis (Fig. 5H).

**Figure 6:**
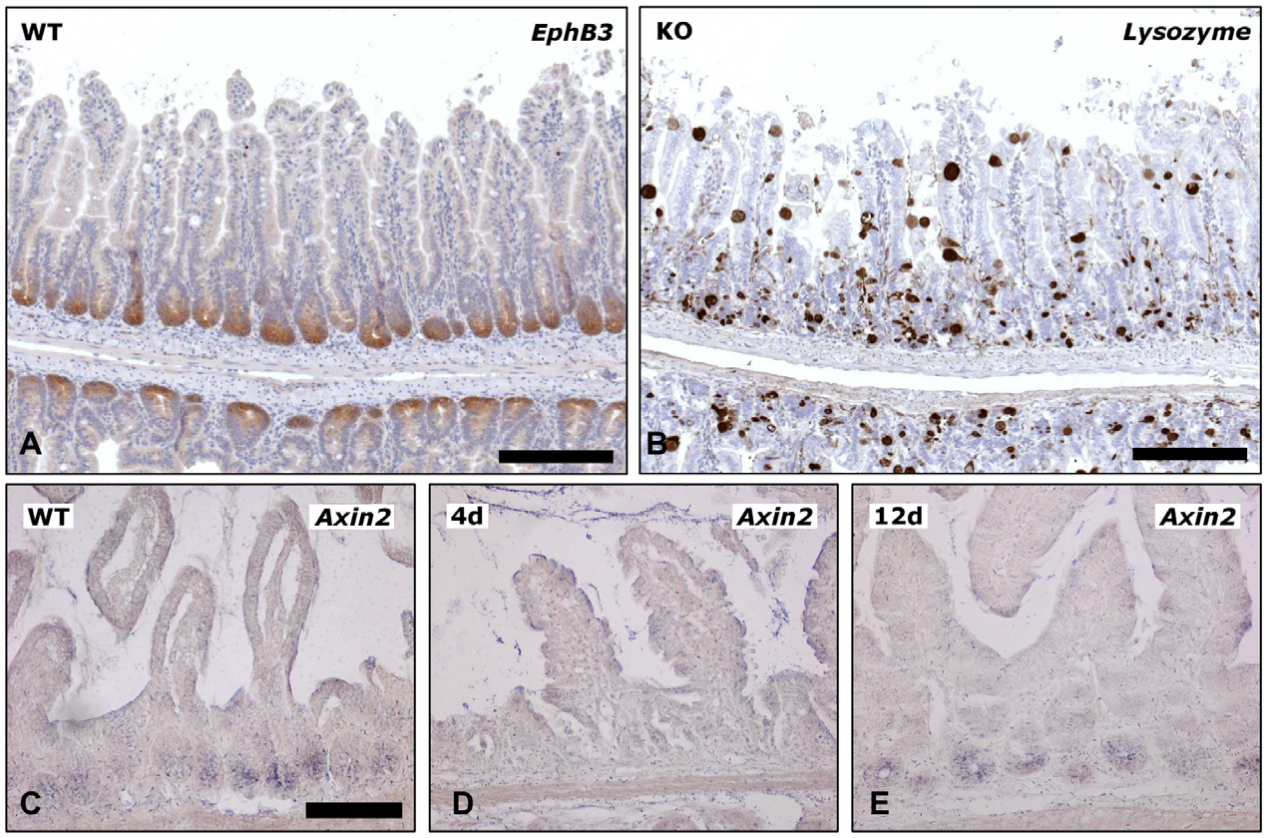
Wnt target gene expression in E-cadherin deficient mice. (A and B) Parallel sections of Cre^+^ *Cdh1* ^fl/fl^ mice analyzed on day 12 after induction of recombination on days 1, 2, 5, and 8 were stained for (A) EphB3 receptor and (B) lysozyme. Bars in A and B, 200 µm. (C–E) In situ hybridization for the Wnt signaling target gene Axin2 (C–E). Strong staining of crypts in control mice (C), loss of target gene expression on day 4 in the short time model (D) and reduced expression in long time model on day 12 (E). Bars in C–E, 100 µm.

Previous studies have indicated that positioning and maturation of Paneth cells depend on Wnt signaling and on the EphB gradient [22], [23]. In induced Cre^+^ *Cdh1* ^fl/fl^ mice staining of the Wnt target gene EphB3 appeared unchanged at the base of the crypts compared to controls (Fig. 6A and not shown). In contrast, on parallel sections in Cre^+^ *Cdh1* ^fl/fl^ mice the lysozyme-positive cells were scattered over the crypts and villi (Fig. 6B). To determine the status of Wnt signaling after loss of E-cadherin in more detail *in situ* hybridization for the target gene Axin2 (Fig. 6C–E) was performed. In control mice, Axin2 is expressed at the base of the crypts (Fig. 6C). On the fourth day following three days of tamoxifen injections expression of Axin2 was almost completely lost at this site (Fig. 6D), indicating loss of Wnt signaling activity at the base of the crypts. The attenuated recombination protocol resulted in retained but reduced target gene expression (Fig. 6E). Of note, irrespectively of the recombination protocol used, no Axin2 positive cells were identified in the upper parts of the crypts or villi indicating absence of active Wnt signaling in the mispositioned Paneth cells. This finding was further confirmed by co-immunostaining for lysozyme and β-catenin: The mispositioned lysozyme-positive cells in the villi revealed strong membraneous but no nuclear β-catenin staining (data not shown).

### Loss of E-cadherin compromises Paneth and goblet cell maturation

**Figure 7:**
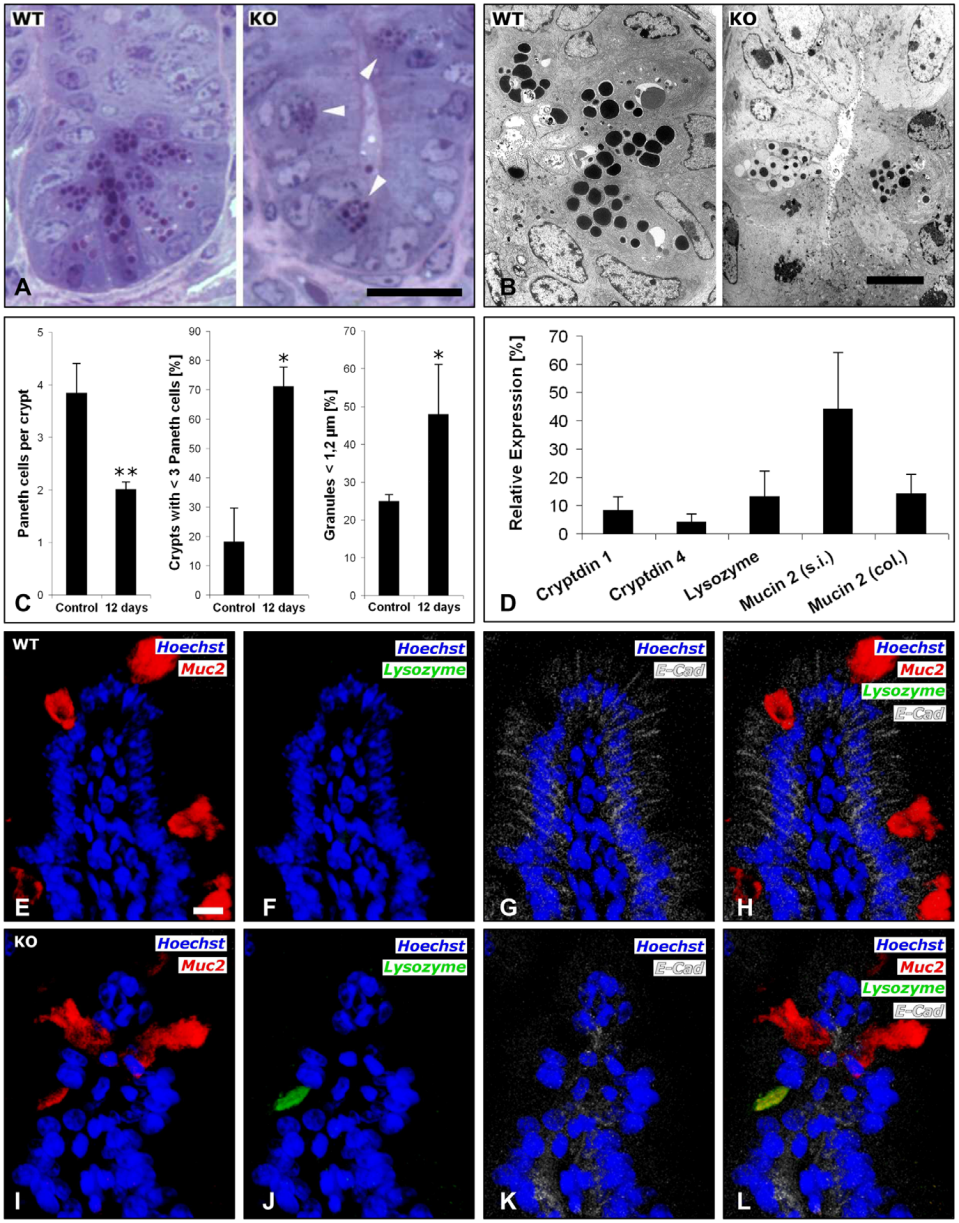
Loss of E-cadherin results in disturbed maturation of Paneth cells. (A–B) Staining of ultra thin sections (A) and transmission electron microscopy (B) revealed a reduced number of Paneth cells at the base of the crypts (arrowheads), altered Paneth cell granule morphology, and reduced numbers and smaller diameters of Paneth cell granules in Cre^+^ *Cdh1* ^fl/fl^ (left panels) compared to control mice (right panels). Bars in A and B, 20 µm and 2.5 µm, respectively. (C) Quantitative analysis of ultra thin sections revealed a reduced number of Paneth cells per crypt and a significant increase in the fraction of cryts with <3 Paneth cells in recombined mice (100 crypts analyzed per mouse for n = 4 mice per group. *, *P*<0.005. Measurement of Paneth cell granules in the same mice revealed an increase in small granules in E-cadherin deficient mice. *P*<0.005. (D) Expression of cryptidin 1 and 4, lysozyme and Muc2 mRNA was determined by quantitative RT-PCR in the intestinal epithelia of control (n = 5) and Cre^+^ *Cdh1* ^fl/fl^ (n = 5) mice. (E–L) Co-immunofluorescence for Muc2 (E, I), lysozyme (F, J), E-cadherin (G, K) in control (E–H) and Cre^+^ *Cdh1* ^fl/fl^ mice (I–L). (H, L) Fusion of pictures. Bars in E–L, 10 µm.

Next, Paneth and goblet cells were studied in more detail. This revealed a strong reduction of Paneth cells residing at the bases of the crypts of Cre^+^ *Cdh1* ^fl/fl^ mice compared to the crypts of control mice (Fig. 7A and B and Fig 7C, left panel). The majority of crypts of Cre^+^ *Cdh1* ^fl/fl^ mice contained less than three Paneth cells per crypt and the remaining Paneth cells were characterized by dysmorphic and pleomorphic granules with a higher amount of small granules (Fig. 7C, right panel). Of note, the large lysozyme and MMP7 positive cells in the villi did not reveal any granules (data not shown). These findings further support the notion of a disturbed maturation of Paneth cells in the recombined mice. To test whether the expression of cryptidines, the most abundant antibacterial product of Paneth cells was reduced, quantitative RT-PCR was performed. This revealed a reduction of cryptidin 1 and cryptidin 4 expression by more than 90% (Fig. 7D; p<0.001 and p<0.001, respectively). As expected from the immunohistochemistry studies, expression of lysozyme was also strongly reduced in the small intestine as was the expression of the most abundant mucin, Muc 2, in both small intestine and colon (Fig. 7D). Co-immunofluorescence studies for Muc2 (Fig. 7E and I), lysozyme (Fig. 7F and J), and E-cadherin (Fig. 7G and K) in control (Fig. 7E–H) and Cre^+^ *Cdh1* ^fl/fl^ mice (Fig. 7I–L) revealed cells in the villi simultaneously expressing markers of Paneth and goblet cells and lacking expression of E-cadherin (Fig.7L). These cells could not be detected in control mice (Fig. 7H) suggesting that they represent a common incompletely differentiated precursor of Paneth and goblet cells.

### Impairment of bacterial defense after loss of E-cadherin

**Figure 8:**
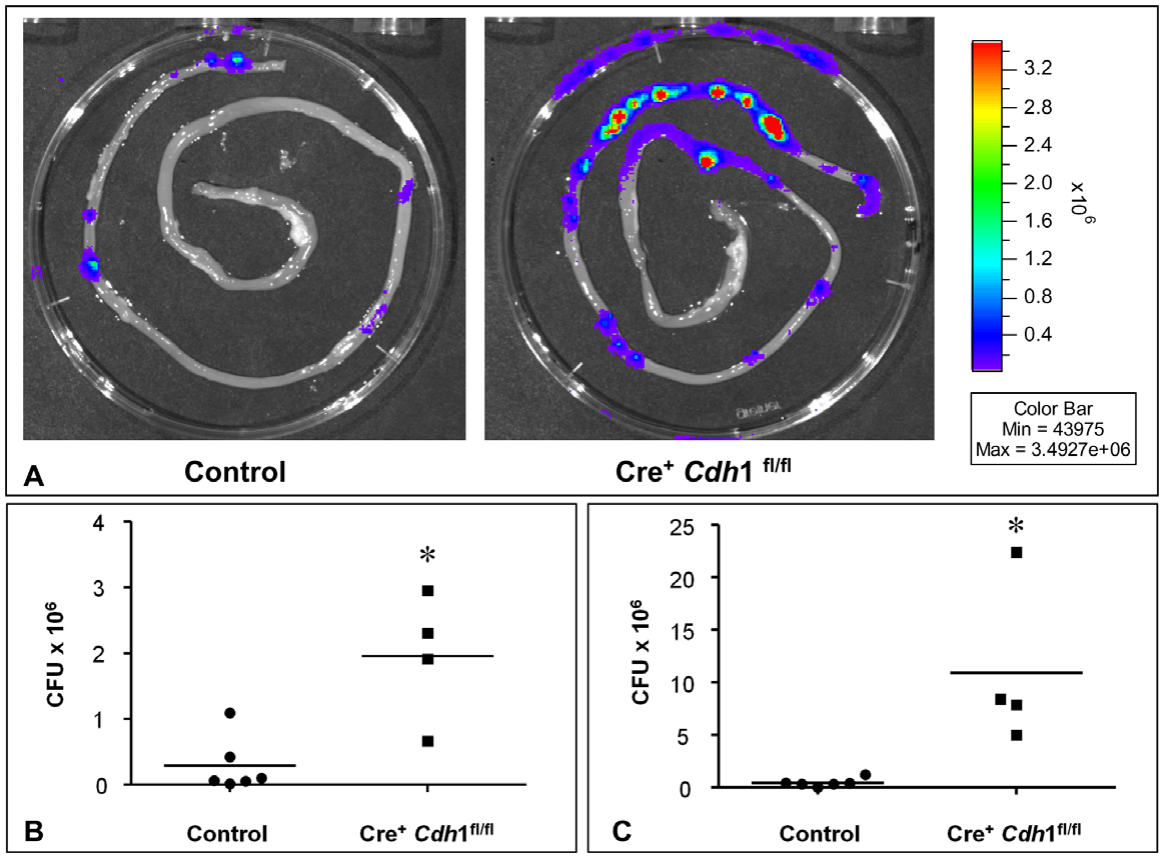
Impairment of bacterial defense in mice deficient for E-cadherin. Increase of bioluminescence in the Peyer's patches in Cre^+^ *Cdh*1^fl/fl^ mice (A, right panel) compared to control mice (A, left panel) 5 days after oral infection with with 10^9^ CFU of bioluminescent *Yersinia enterocolitica*. Colonization of small intestine (B), and Peyer's Patches (C) of Cre^+^ *Cdh1* ^fl/fl^ and control mice (Cre^+^ *Cdh1* ^wt/fl^). CFU for each mouse is shown and bars represent the median value. The limit of detection for yersiniae was 10 CFU for Peyer's Patches and 50 CFU for small intestine. Statistical significance is indicated by asteriks.

Mucus-producing goblet cells and Paneth cells which produce a wide array of antimicrobial substances are involved in the first line of bacterial defense. Based on our findings we speculated that Cre^+^ *Cdh1* ^fl/fl^ mice might have acquired a deficiency in clearing pathogenic bacteria from the intestinal lumen. To test this hypothesis, Cre^+^ *Cdh1* ^fl/fl^ mice and control mice were induced according to the milder tamoxifen protocol and were orally fed with 10^9^ colony forming units (CFU) of bioluminescent *Yersinia enterocolitica* as a model for enteropathogenic bacteria on day nine after start of recombination. Analysis 5 days after infection revealed a strong increase of bioluminescence in the Peyer's patches in the Cre^+^ *Cdh*1^fl/fl^ mice compared to controls (Fig. 8A). The luminal bacterial load in small intestine was significantly higher in Cre^+^ *Cdh*1^fl/fl^ than in control mice (median number of colony forming yersiniae, 2.11×10^6^ vs. 8.00×10^4^; p = 0.019; Fig. 8B). Moreover, Peyer's patches were colonized by significantly higher numbers of yersiniae in Cre^+^ *Cdh*1^fl/fl^ mice compared to control mice (median number of colonies, 8.14×10^6^ vs. 3.51×10^5^; p = 0.01; Fig. 8C).

## Discussion

Our findings demonstrate that E-cadherin has an essential role in intestinal homeostasis and differentiation of secretory cell lineages. E-cadherin is required for the maintenance of architecture and function of the intestinal epithelium. Cellular adhesion and regulation of Paneth and goblet cell positioning and maturation is dependent on the proper expression of E-cadherin. Therefore, E-cadherin has a key role in maintaining intestinal epithelial integrity and is indirectly involved in the defense against enteropathogenic bacteria.

The main function of Paneth cells is the secretion of microbicidal peptides such a α-defensins which is critical in the defense against enteropathogenic bacteria [24]. Paneth cells appear during early postnatal life and they differentiate while migrating towards the crypt base, where they reside for about 20 days before being cleared by phagocytosis [25]. Maturation and positioning of Paneth cells has previously been linked to Wnt signaling [26]–[29]. It has been suggested that the β-catenin target gene PPARβ/δ is an important mediator of Wnt activity in Paneth cells [30]. Targeted deletion of β-catenin or its target genes Fz-5 or EphB3 resulted in random scattering of Paneth cells throughout the crypt-villus axis similar to our findings [22], [26], [31], [32]. Our data reveal that E-cadherin is required for correct maturation and placement of Paneth cells, supporting its role in cell polarization [6]. Interestingly, this occurs without perturbation of the Ephrin/EphB system. Loss of E-cadherin might either result in a defect in cell sorting allowing common Paneth and goblet cell precursors to migrate along the crypt-villus axis where they are no longer exposed to Wnt signals coinciding with impaired maturation, or E-cadherin might be required for anchoring and maturation of Paneth cells at the base of the crypts. Alternatively, adequate E-cadherin levels may be a prerequisite for the appropriate differentiation from stem cells towards goblet and Paneth cells.

Our work also reveals the importance of E-cadherin for the maintenance of intestinal epithelial homeostasis. The first event in lineage determination of a progenitor stem cell has been attributed to Notch signaling. Math 1 (mouse atonal homolog 1) is thought to be the master switch for differentiation into the two principal lineages, the absorptive enterocytes and the secretory cells [33], [34]. Low levels of Math-1 lead to differentiation of cells towards the enterocyte lineage, while cells with sustained Math1 expression develop towards the three secretory cell lineages, Paneth, goblet, and enteroendocrine cells [3]. For further specification into enteroendocrine cells the two basic helix-loop-helix factors neurogenin 3 and NeuroD1 are required [35], [36], while Rac1 participates in the fate decision of Math1-positive cells toward Paneth and goblet cells [37]. The *de novo* appearance of cells in the villi of recombined mice which can be detected by Paneth cell and goblet cell stainings is compatible with the notion that these cells are common incompletely differentiated precursors of Paneth and goblet cells. Moreover, these cells fail to stain positive for Wnt target genes as would be expected in mature Paneth cells. The simultaneous appearance of incompletely differentiated, mispositioned cells and of normally differentiated cells is most likely due to the coexistence of E-cadherin deficient and E-cadherin expressing cells owing to incomplete recombination in the extended tamoxifen injection protocol. Interestingly, E-cadherin has been shown to recruit Rac1 which in turn leads to stabilization of the anchoring of the E-cadherin-catenin complex to actin [38]. Therefore, loss of E-cadherin expression is likely to result in the dissociation of Rac1 which may explain the disturbed maturation and positioning of Paneth and goblet cells observed.

E-cadherin's function in epithelial homeostasis has probably been best studied in skin [39]–[41]. Here, loss of E-cadherin resulted in death of mice shortly after birth due to dehydration [41], altered differentiation [39], and disturbed epidermal integrity [40]. Interestingly, E-cadherin was found to be required for tight junction formation but not desmosome assembly in skin [41]. This stands in contrast to our findings, where loss of E-cadherin does not affect the ultrastructure of tight junctions, but results in loss of desmosomes. This role of E-cadherin for the assembly of other junctional complexes including desmosomes, tight junctions, and gap junctions [42] has been suggested to be related to the function of adherens junctions to establish cell polarity [41]. However, the reason for this difference observed between skin and intestine is not clear. It may be due to the relatively short period of time between start of recombination and analysis of mice in our model and the fact that loss of E-cadherin occurred in the adult mouse and not during embryogenesis.

Apoptosis occurred earlier in the small intestine than in the colon. This is well explained by the stronger activity of the Cre recombinase in small intestine [17]. However, the epithelial defect, including sheets of cells shed into to lumen, was more severe in the colon than in the small intestine. In colon, loss of the entire crypt epithelium was observed with only the surface epithelium remaining, in small intestine the phenotype was milder with a reduction of staining intensity for E-cadherin of the crypt-villus axis. This suggests a yet unexplained stronger dependence of the colon epithelium on E-cadherin-dependent cell adhesion than the small intestine. The stronger effect in colon was also true for the milder recombination protocol: In colon, expression of E-cadherin was almost completely lost, whereas in small intestine, expression was strongly reduced but not lost. Previous *in vitro* [43] and *in vivo* [14] studies have suggested an anti-apoptotic function of E-cadherin in the intestine. A mouse model expressing a dominant negative N-cadherin mutant in the mouse intestine resulted in an increase in epithelial apoptosis rates, increased migration of enterocytes along the crypt-villus axis and impaired cellular differentiation [14], [44]. These mice displayed a decrease and intracellular redistribution of E-cadherin, suggesting a role for E-cadherin in intestinal homeostasis. But as expression of a dominant negative N-cadherin also interferes with fibroblast growth factor receptor activation and mitogen-activated protein kinase-extracellular signal-regulated kinase (MAPK-ERK) signaling [45], [46], the precise role of E-cadherin in this context remained unclear. Another study by the same authors analyzed mouse intestinal epithelium over-expressing of E-cadherin [47]. Here proliferation was suppressed, apoptosis was induced, and movement along the crypt-villus axis was reduced. Targeted deletion of the E-cadherin interacting protein p120-catenin led to a disrupted intestinal barrier and impaired epithelial homeostasis [15]. Therefore, our study by directly targeting E-cadherin now confirms and expands these previous indirect findings and further clarifies E-cadherin's critical role as an anti-apoptotic and survival factor of the intestinal epithelium.

Interestingly, when it colonizes the intestine, *Bacteroides fragilis* can produce the enterotoxin fragilysin which acts as a protease that disrupts adherens junctions by proteolysis of the extracellular domain of E-cadherin [48] . This results in an increase in paracellular permeability and morphological changes of intestinal epithelial cells [49]. Thus, the effects observed in our model might resemble the effects of *B. fragilis* on the intestinal epithelial lining. Intriguingly, *B. fragilis* is responsible for >60% of the biofilm mass in patients with Crohn's disease but only for 16% of the biofilm mass in healthy controls [50]. Moreover, *B. fragilis* has been found to be one of the predominant bacteria isolated from abdominal abscesses of patients with Crohn's disease [51]. The reduced expression of Paneth cell α-defensins has been described in ileal Crohn's disease [52] and expression analyses in human Crohn's disease biopsies have demonstrated down-regulation of E-cadherin [8]–[10]. Therefore, it has been suggested that Paneth cell deficiency and disturbed epithelial barrier function are host risk factors for the development of Crohn's disease [53], [54]. Our results demonstrate that these two mechanisms of host defense are both dependent on E-cadherin.

In conclusion, we provide evidence for an essential role of E-cadherin in intestinal homeostasis. E-cadherin functions as a survival factor for the small intestinal and colonic epithelium and its expression is a prerequisite for the maintenance of the epithelial defense function. These findings further our understanding of the role of E-cadherin in the pathogenesis of diseases of the intestine including chronic inflammatory bowel disease and infections with enteropathogenic bacteria.
